# Association of Spinal Cord Stimulator Implantation With Persistent Opioid Use in Patients With Postlaminectomy Syndrome

**DOI:** 10.1001/jamanetworkopen.2021.45876

**Published:** 2022-01-31

**Authors:** To-Nhu Vu, Chachrit Khunsriraksakul, Yakov Vorobeychik, Alison Liu, Renan Sauteraud, Ganesh Shenoy, Dajiang J. Liu, Steven P. Cohen

**Affiliations:** 1 Department of Anesthesiology and Perioperative Medicine, Penn State College of Medicine, Hershey, Pennsylvania; 2 Penn State College of Medicine, Hershey, Pennsylvania; 3 Department of Public Health Sciences, Penn State College of Medicine, Hershey, Pennsylvania; 4 Departments of Public Health Sciences and Biochemistry & Molecular Biology, Penn State College of Medicine, Hershey, Pennsylvania; 5 Departments of Anesthesiology and Critical Care Medicine, Physical Medicine & Rehabilitation, Neurology, and Psychiatry and Behavioral Sciences, Johns Hopkins School of Medicine, Baltimore, Maryland; 6 Departments of Anesthesiology and Physical Medicine & Rehabilitation, Walter Reed National Military Medical Center, Uniformed Services University of the Health Sciences, Bethesda, Maryland

## Abstract

**Importance:**

The results of studies evaluating spinal cord stimulation (SCS) for postlaminectomy syndrome (PLS) have yielded mixed results. This has led to an increased emphasis on objective outcome measures such as opioid prescribing.

**Objective:**

To determine the association between SCS and long-term opioid therapy (LOT) for PLS.

**Design, Setting, and Participants:**

In this cohort study, adults with PLS were identified using the TriNetx Diamond Network and separated based on whether they underwent SCS. Patients were stratified according to baseline opioid use (opioid-naive or receiving LOT) and subsequent opioid therapy over the 12-month period ranging from 3 to 15 months post-SCS implantation or post-PLS index date. Statistical analysis was performed from June to December 2021.

**Exposure:**

SCS.

**Main Outcomes and Measures:**

The main outcome was cessation of opioid use among patients receiving LOT or abstinence from opioids among opioid-naive patients. Opioid-naive patients were defined as those receiving at most 2 opioid prescriptions per year, and patients on LOT were those receiving at least 6 opioid prescriptions per year.

**Results:**

Among 552 937 eligible patients treated between December 2015 and May 2021, 26 179 with PLS received an SCS implant. The median (IQR) patient age was 60 (51-69) years; 305 802 patients (55.3%) were female. Among those reporting racial identify (37.0% [204 758 patients]), 9.3% (18 971 patients) were African American, 0.3% (648 patients) were Asian, and 90.4% (185 139 patients) were White. Compared with those who did not receive an SCS, individuals who received an SCS were more likely to be using opioids preimplantation (mean [SD] prescriptions: 4.3 [8.5] vs 4.1 [9.3]; *P* < .001) but less likely to be using opioids after SCS implantation (mean [SD] prescriptions: 3.8 [8.2] vs 4.0 [9.4]; *P* = .006). In the 12-month study period, similar proportions in the SCS and no-SCS groups receiving baseline LOT remained on LOT (70.3% [n = 74 585] vs 69.2% [n = 3882], respectively; *P* = .10). In opioid-naive patients, SCS was associated with a small decreased likelihood of patients subsequently receiving LOT (7.6% vs 7.0%; difference, −0.6% [95% CI, −1.0% to −0.2%]; *P* = .003). In multivariable analysis, SCS was associated with an increased likelihood of not being on opioids in both opioid-naive (adjusted odds ratio [OR], 0.90 [95% CI, 0.85-0.96]; *P* < .001) and LOT patients (adjusted OR, 0.93 [95% CI, 0.88-0.99]; *P* = .02). White patients were significantly more likely to be diagnosed with PLS (ie, underwent surgery) (90.4% vs 85.2%; difference, 5.2% [95% CI, 5.1%-5.4%]; *P* < .001) and receive an SCS (93.7% vs 90.3%; difference, 3.4% [95% CI, 2.9% to 4.0%]; *P* < .001) than patients of other racial identities.

**Conclusions and Relevance:**

These findings suggest that under real-life conditions, SCS was associated with small, clinically questionable associations with opioid discontinuation and not starting opioids in the context of PLS.

## Key Points

**Question:**

What is the association between spinal cord stimulation and long-term opioid use in patients with postlaminectomy syndrome?

**Findings:**

In this large cohort study involving 552 937 patients treated between 2015 and 2021, spinal cord stimulation was associated with a statistically significant reduction in opioid use in both opioid-naive patients and in those on long-term opioid therapy (LOT) in multivariable, but not univariable, analysis. However, the relevance of the reduction is clinically questionable.

**Meaning:**

These findings suggest that in real-life conditions, spinal cord stimulation may not result in clinically meaningful cessation of LOT but may be associated with a lower rate of new-onset LOT use.

## Teaser

This cohort study examines the association between spinal cord stimulation treatment and long-term opioid therapy among patients with postlaminectomy syndrome (PLS).

## Introduction

Low back pain (LBP) is the leading cause of disability worldwide, with point and 1-month prevalence rates of 12% and 23%, respectively.^1,2^ Each year in the US, approximately 900 000 patients undergo spine surgery, with the incidence of failed back surgery syndrome, also called postlaminectomy syndrome (PLS), being around 19% for laminectomy and 46% for fusion.^3,4,5^

The treatment of PLS is challenging. The use of spinal cord stimulation (SCS) to treat PLS, the most common indication, has surged dramatically with the advent of new technologies that purport to better alleviate axial pain.^6^ The global SCS market size is expected to reach $2.8 billion by 2025, with an anticipated growth rate of 8.3% per year.^7,8^ Despite high reported success rates in the literature, the quality of studies is low, with industry-sponsored trials reporting better outcomes than non-industry-sponsored studies.^6^ This discrepancy may be due to methodological flaws and bias, short follow-up periods, and lack of objective outcome measures.

One objective outcome that has attracted attention is opioid consumption. From 1999 to 2010, the proportion of visits to ambulatory-based physicians for spinal pain in which an opioid was prescribed increased from 19% to 29%, with one review finding that 42% of patients with LBP received an opioid prescription within a year of diagnosis.^9,10^ In a systematic review evaluating the ability of SCS to reduce opioid consumption in patients with back and/or limb pain, Pollard et al^11^ found that SCS significantly decreased opioid use.^11^ However, this review included only a small percentage of randomized SCS trials, and individuals enrolled in the mostly industry-sponsored studies included may be more incentivized to reduce opioid consumption than the general population. A prior database study found that among 8497 patients who underwent SCS placement between 2010 and 2015, 60.4% experienced some reduction in their opioid use, 34.2% reduced their dose by a clinically meaningful amount, and 17.0% discontinued opioids entirely.^12^ In this analysis, more than one-quarter of patients were excluded due to loss of insurance, explantation within 1 year of surgery, and use of very high doses of opioids after implantation.

To determine the association between SCS and opioid use in patients with PLS in clinical practice, we analyzed opioid prescribing patterns in patients who underwent subsequent SCS implantation using the TriNetX Diamond Network. Our objectives were to: (1) determine the association of SCS with the cessation of long-term opioid therapy (LOT) compared with absence of SCS; (2) determine the association between SCS and subsequent LOT in opioid-naive patients; and (3) determine the association demographic and clinical variables have with opioid use in PLS patients and how these variables interact with SCS opioid outcomes. Given the multiple reports of SCS contributing to the opioid-use reduction for chronic pain,^13,14,15,16^ we hypothesized that SCS would be inversely associated with opioid use.

## Methods

This cohort study received approval as an exempt protocol from the Penn State University Office for Research Protections on the basis of deidentified and publicly available data. This study follows the Strengthening the Reporting of Observational Studies in Epidemiology (STROBE) reporting guideline for observational studies.

### Data

The TriNetX Diamond platform was accessed between May and August 2021 for eligible patients treated between December 2015 and May 2021. Patients with a PLS diagnosis (N = 1 165 723) were identified based on *International Classification of Diseases, Ninth Revision (ICD-9) *and* ICD-10* codes (eTable 1 in the Supplement) using the TriNetX Diamond Network, a proprietary platform allowing access to aggregate-level deidentified data for patients in 99% of US health plans that includes diagnoses and prescribed medications for more than 200 million patients. Per TriNetX research administrators, opioid medication ingredient records are an accurate surrogate for prescriptions (personal email communication on August 18, 2021, from Seth Kuranz, PhD, Director of Clinical Sciences, TriNetX).

### Selection Criteria and Spinal Cord Stimulation Status

Detailed descriptions regarding cohort definitions can be found in eFigure 1 in the Supplement. For cases, we identified patients receiving SCS based on *ICD-9* and *ICD-10* codes (eTable 1 in the Supplement) and defined the index date as the date of SCS implantation. Included in the case group were patients who were at least 18 years of age at the time of SCS implant and those who remained within a participating health care organization for 12 months prior and 15 months after the index date. When patients who underwent SCS prior to their PLS diagnosis and those who underwent spine surgery within the range of 12 months before and 15 months after the index date were excluded, there were 35 204 patients remaining. To reduce the effect of pain duration which has been shown to be associated with poorer prognosis, we further excluded patients who received an SCS over 18 months (corresponding to the third quartile of time between PLS diagnosis and SCS implant) after a PLS diagnosis, resulting in 26 179 patients in the case group (eFigure 1 in the Supplement).

Because PLS diagnoses always preceded SCS implantation, we accounted for possible differences in disease burden (greater in patients with SCS on LOT who had longer duration of symptoms, lower in opioid-naive patients with SCS who might have had PLS for longer time periods without opioids) by randomly assigning an index date after PLS diagnosis in the control group that matched the distribution of days between PLS diagnosis and SCS placement in the case group. Included in the control group were patients who were at least 18 years of age at the adjusted index date and those who remained within a participating health care organization between 12 months before and 15 months after the index date. When patients who underwent spine surgery within the range of 12 months before and 15 months after the adjusted index date were excluded, 526 758 patients remained in the control group.

### Classification of Opioid Users and Primary End Point

We classified patients into categories based on our primary outcome measure, cessation of (in patients on LOT) or abstinence from (in opioid-naive patients) opioid use at the primary end point. Opioids surveilled were identified using RxNorm codes and included hydromorphone, hydrocodone, oxymorphone, oxycodone, fentanyl, and morphine, whereas those excluded were opioids combined with nonanalgesic substances (eg, antihistamines), mixed mechanism opioids (tramadol, tapentadol), and opioids with nonanalgesic indications (buprenorphine, methadone) (eTable 2 in the Supplement). At baseline, we defined opioid-naive patients as those who received at most 2 opioid prescriptions in the year prior to PLS index date or an SCS implant to account for prescriptions written for nonspinal indications (eg, procedures, injuries), whereas LOT was defined as receiving at least 6 opioid prescriptions in this time frame. At the primary end point, we defined patients on LOT as those who received at least 6 opioid prescriptions within the 12-month period of 3 to 15 months after their PLS index date and/or SCS implant to control for opioid prescriptions provided in the 3-month postsurgical period and because many patients receive more than a 1-month supply of opioid medications in a single prescription (particularly with out-of-state telehealth) or receive prescriptions from out-of-network clinicians during vacations or stays at secondary residences.^17^ To reduce bias, we also performed propensity score matching and applied more stringent (≥10) and liberal (≥4 prescriptions in the study time frame) designations of LOT which were compared with the principal definition in sensitivity analyses.

### Data Collection

Besides SCS and opioid-use status, the following data were collected to determine their association with baseline and post-SCS or post-PLS-diagnosis opioid status and whether they received an SCS: age, sex, race, psychosocial history, medical comorbidities (individually and by Charlson comorbidity index score; eTable 3 in the Supplement), and contemporaneous medications that elevate the risk of opioid therapy.^18^ Demographic information including race, ethnicity, and sex is entered into the TriNetX database from electronic medical records, which is usually but not always patient-reported.

### Outcome Measures

The primary outcome measure was the percentage of patients with PLS on LOT after SCS vs no SCS 3 to 15 months after the index date. Subgroup analyses of previously opioid-naive vs patients on LOT were performed. The secondary outcome measure was change in mean number of opioid prescriptions after SCS vs no SCS. Exploratory analyses were done to determine which factors were independently associated with LOT and receiving an SCS.

### Statistical Analysis

Statistical analyses were conducted using R version 3.6 (R Project for Statistical Computing) from June to December 2021. Descriptive statistics are presented as proportions for categorical variables and as means and SDs for continuous variables. χ^2^ test was used to assess the statistical significance of differences between proportions for categorical variables, whereas the *t* test was used to assess differences for continuous variables. Demographic and clinical variables evaluated for their association with opioid use were chosen based on literature demonstrating a relationship between opioid prescribing and treatment response rate. In the multivariable logistic regression model assessing the likelihood of a patient receiving SCS, we included age, Charlson comorbidity index score, sex, race, psychosocial risk factors, and psychotropic medication use. For multivariable logistic regression assessing LOT, SCS status was included in the model. Multivariable regression results are presented as adjusted odd ratios (aORs) with 95% CIs. To account for possible confounding by indication (ie, physicians selectively offer SCS implantation to patients likely to benefit from it), we performed a propensity score matching analysis. To create a matched data set between patients with and without SCS, we used similar covariates as in multivariable logistic regression and defined our distance with logistic regression using the nearest neighbor method and selected matches within a caliper distance of 0.2 SD of the logit propensity score, with a matching ratio of 1:10 without replacement. Missing demographic data were either noted (eg, unknown race) or dropped. Two-sided statistical tests were considered significant for *P* < .05.

## Results

### Demographic Data

**Figure 1.**
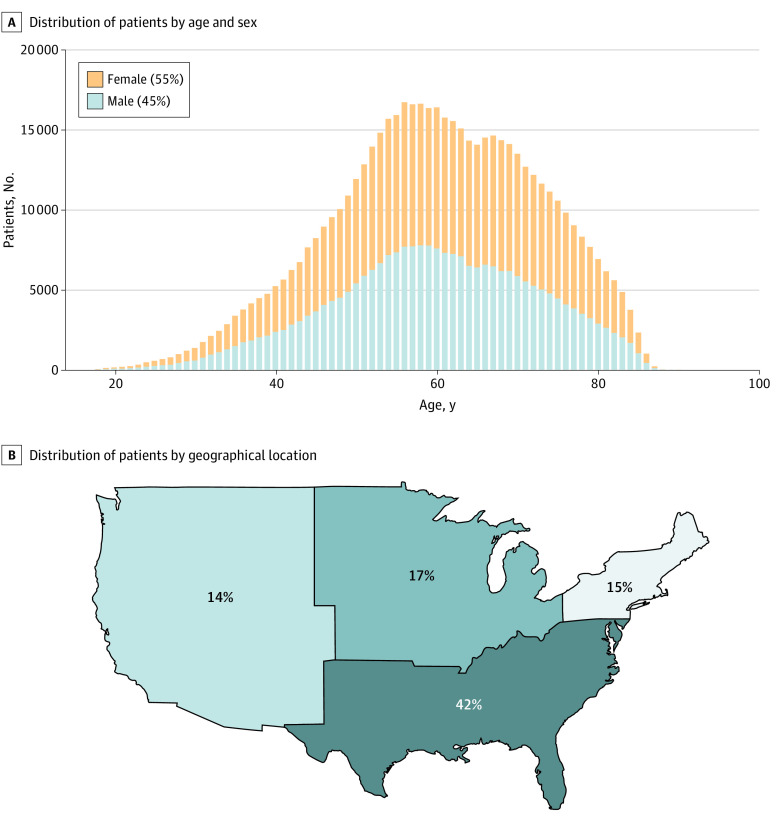
Age, Sex, and Geographic Distribution of Eligible PLS Patients (N = 552 937) Within the TriNetX Diamond Network PLS indicates postlaminectomy syndrome.

Among 1 165 723 patients with a diagnosis of PLS (including fusion and instrumentation) whose records were reviewed, 552 937 patients were eligible for inclusion (26 179 patients [4.7%] with a SCS and 526 758 patients [95.3%] without). For baseline opioid use status, 384 552 patients were opioid-naive and 123 553 were on LOT. The median (IQR) duration between PLS diagnosis and SCS placement was 2.3 (0.2-6.7) months. Figure 1 shows demographic data among study patients. The median (IQR) age of patients was 60 (51-69) years, with 305 802 patients (55.3%) being female. Among those reporting racial identify (37.0% [204 758 patients]), 9.3% (18 971 patients) were African American, 0.3% (648 patients) were Asian, and 90.4% (185 139 patients) were White. Compared with overall data from the Diamond Network, patients receiving a PLS diagnosis were more likely to be White (90.4% vs 85.2%; difference, 5.2% [95% CI, 5.1%-5.4%]; *P* < .001) than African-American (9.3% vs 12.8%; difference, −3.5% [95% CI, −3.5% to −3.7%]; *P* < .001) or Asian (0.3% vs 2.0%; difference, −1.7% [95% CI, −1.7% to −1.6%]; *P* < .001). Compared with PLS patients not receiving an SCS, PLS patients who received an SCS were also more likely to be White (93.7% vs 90.3%; difference, 3.4% [95% CI, 2.9% to 4.0%]; *P* < .001) than African American (6.1% vs 9.4%; difference, −3.3% [95% CI, −3.8% to −2.8%]; *P* < .001) or Asian (0.2% vs 0.3%; difference, −0.1% [95% CI, −0.3% to −0.1%]; *P* = .01). Other factors associated with receiving an SCS implant included older mean (SD) age (61.38 [12.94] years vs 59.77 [12.72] years; *P* < .001), female sex (56.5% vs 55.2%; difference, 1.3% [95% CI, 0.7% to 1.9%]; *P* < .001), higher mean (SD) Charlson comorbidity index scores (1.21 [1.80] vs 1.15 [1.79]; *P* < .001), presence of psychosocial risk factors, and use of psychotropic medications (eTable 4 in the Supplement). Unlike in univariate analysis, the results of multivariable analysis found that patients with a history of antipsychotic use (OR, 0.92 [95% CI, 0.87-0.98]; *P* = .01) were less likely to receive SCS. There was no significant association between female sex and SCS implantation (eFigure 2 in the Supplement). Although TriNetX includes data from health care organizations (HCOs) across the United States, a substantial plurality of patients were from the South (41.3% [292 333 patients]).

### Association of SCS With Long-term Opioid Therapy

**Figure 2.**
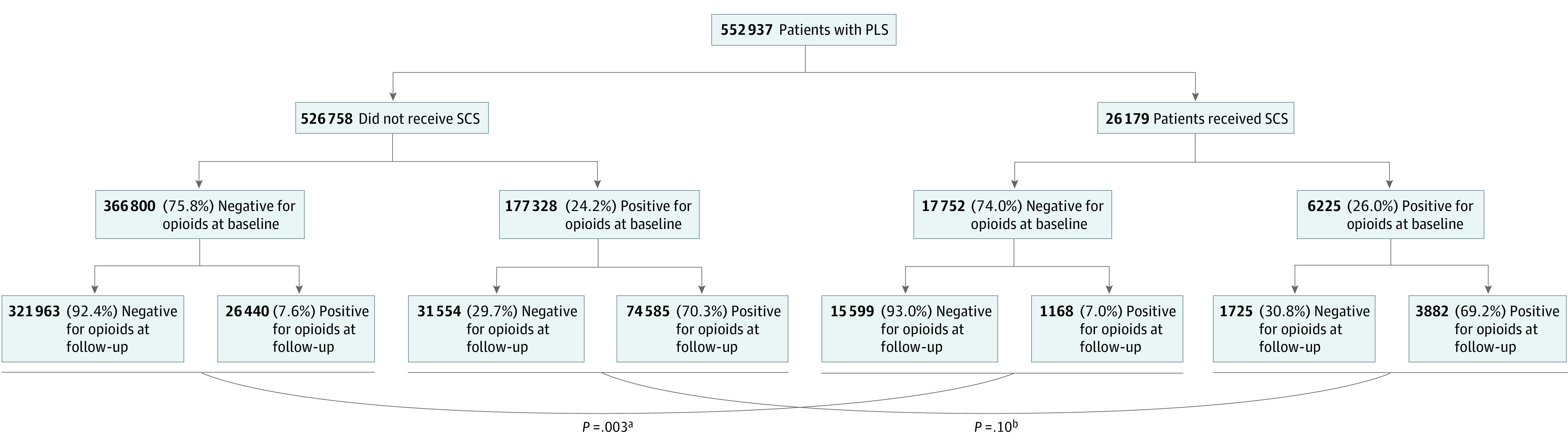
Breakdown of PLS Cohort by SCS Status, Baseline Opioid Status, and Opioid Use Status at 3 to 15 Months After Adjusted Index Date (for Patients Without SCS) or SCS Implant in Diamond Network Long-term opioid therapy is defined as at least 6 prescriptions per year. For baseline opioid status, positive is defined as at least 6 prescriptions per year and negative is defined as at most 2 prescriptions per year. For opioid use status, positive is defined as at least 6 prescriptions per year and negative is defined as at most 2 prescriptions per year. PLS indicates postlaminectomy syndrome; SCS indicates spinal cord stimulation. ^a^*P* = .003 refers to the statistical significance between opioid-native patients who remained abstinent from long-term opioids vs starting long-term opioid therapy, stratified by SCS implantation or no SCS implantation. ^b^*P* = .10 refers to the statistical significance between patients on baseline long-term opioid therapy who remained on long-term opioid therapy vs discontinuing long-term opioids, stratified by SCS implantation or no SCS implantation.

We stratified patients in each group according to baseline opioid use status. Defining opioid-naive patients as those receiving at most 2 opioid prescriptions per year and patients on LOT as those who received at least 6 opioid prescriptions per year, we found that SCS resulted in a significantly decreased likelihood of patients requiring LOT only in patients who were previously opioid-naive (7.6% vs 7.0%; difference, −0.6% [95% CI, −1.0% to −0.2%]; *P* = .003) (Figure 2). This finding was replicated only when the more stringent definition of LOT (≥10 opioid prescriptions per year) was applied (eFigure 3 in the Supplement). Among patients receiving LOT before the PLS index date (26.0% [n = 6225] of SCS patients vs. 24.2% [n = 117 328] of non-SCS patients), similar proportions of the SCS (69.2% [n = 3882]) and non-SCS (70.3% [n = 74 585]) groups remained on LOT (difference, −1.1% [−2.3% to 0.2%]; *P* = .10).

Table 1 shows the characteristics associated with LOT in opioid-naive patients with PLS. Opioid-naive patients who later went on LOT were more likely to be younger (mean [SD] age: 57.56 (12.12) years vs 60.98 (12.89) years; *P* < .001), female (57.2% vs 54.9%; difference, 2.3% [95% CI, 1.7% to 2.9%]; *P* < .001), White (40.0% vs 30.7%; difference, 9.3% [95% CI, 8.7% to 9.9%]; *P* < .001), have lower Charlson comorbidity index scores (1.12 [1.77] vs 1.15 [1.78]; *P* = .004), did not receive an SCS (4.2% vs 4.6%; difference, −0.4% [95% CI, −0.6% to −0.1%]; *P* = .003), have myriad positive psychosocial risk factors, and use psychotropic medications.

**Table 1.** Characteristics of Opioid-Naive Patients With Postlaminectomy Syndrome in Diamond Network Stratified by Opioid Use Status 3 to 15 Months After Postlaminectomy Syndrome Diagnosis (for Patients Without SCS) or SCS Implant

Characteristic	Patients, No. (%)		*P* value
	Opioid-naive (n = 337 562)^a^	Long-term opioid use (n = 27 608)^b^	
**Variables**			
Continuous, mean (SD)			
Age at index event, y	60.98 (12.89)	57.56 (12.12)	<.001
Charlson comorbidity index score	1.15 (1.78)	1.12 (1.77)	.004
Categorical			
SCS			
Yes	15 599 (4.62)	1168 (4.23)	.003
No	321 963 (95.38)	26 440 (95.77)	
Sex			
Female	185 199 (54.86)	15 785 (57.18)	<.001
Male	152 241 (45.10)	11 819 (42.81)	
Race			
African American	10 737 (3.18)	1140 (4.13)	<.001
Asian	453 (0.13)	27 (0.10)	
White	103 603 (30.69)	11 033 (39.96)	
Unknown	222 769 (65.99)	15 408 (55.81)	
Psychosocial history^c^			
Smoking	65 434 (19.38)	7200 (26.08)	<.001
Alcohol abuse	6214 (1.84)	579 (2.10)	.003
Nonalcohol substance use disorder	35 648 (10.56)	4323 (15.66)	<.001
Long-term opioid use	0	0	NA
Depression diagnosis	59 451 (17.61)	6127 (22.19)	<.001
Anxiety diagnosis	44 129 (13.07)	4706 (17.05)	<.001
Psychosis diagnosis	5297 (1.57)	559 (2.02)	<.001
Current medications^c^			
Antidepressant use	40 464 (11.99)	5999 (21.73)	<.001
Benzodiazepine use	31 535 (9.34)	4698 (17.02)	<.001
Antipsychotic use	5865 (1.74)	1065 (3.86)	<.001
Systemic comorbidities^d^			
Myocardial infarction	12 195 (3.61)	1020 (3.69)	.49
Congestive heart failure	20 085 (5.95)	1632 (5.91)	.80
Peripheral vascular disease	32 928 (9.75)	2131 (7.72)	<.001
Cerebrovascular disease	30 694 (9.09)	2137 (7.74)	<.001
Dementia	3061 (0.91)	161 (0.58)	<.001
Chronic pulmonary disease	78 631 (23.29)	7076 (25.63)	<.001
Rheumatic disease	17 866 (5.29)	1481 (5.36)	.62
Peptic ulcer disease	7890 (2.34)	759 (2.75)	<.001
Liver disease	20 578 (6.10)	1823 (6.60)	.001
Diabetes	79 472 (23.54)	6105 (22.11)	<.001
Hemiplegia/paraplegia	5931 (1.76)	484 (1.75)	.98
Kidney disease	23 936 (7.09)	1583 (5.73)	<.001
Any malignant neoplasm	22 422 (6.64)	1391 (5.04)	<.001
Metastatic solid tumor	3598 (1.07)	340 (1.23)	.01
AIDS/HIV	1011 (0.30)	96 (0.35)	.18

Abbreviation: SCS, spinal cord stimulation.

^a^ Opioid-naive is defined as at most 2 prescriptions per year.

^b^ Long-term opioid use is defined as at least 6 prescriptions per year.

^c^ 1-year prior to index date.

^d^ Any time prior to index date.

Table 2 illustrates the characteristics associated with LOT in patients with PLS already on LOT. Patients who remained on LOT were more likely to be older (mean [SD] age: 58.02 [11.62] years vs 57.28 [12.65] years; *P* < .001), female (56.4% vs 54.3%; difference, 2.1% [95% CI, 1.4% to 2.7%]; *P* < .001), have lower mean (SD) Charlson comorbidity index scores (1.12 [1.76] vs 1.15 [1.81]; *P* = .04), and lack prior psychosocial risk factors, but use psychotropic medications.

**Table 2.** Characteristics of Patients With Postlaminectomy Syndrome Prescribed Long-term Opioid Therapy in Diamond Network Stratified by Opioid Use Status 3 to 15 Months After Postlaminectomy Syndrome Diagnosis (for Patients Without SCS) or SCS Implant

Characteristic	Patients, No. (%)		*P* value
	Opioid-naive (n = 33 279)^a^	Long-term opioid use (n = 78 467)^b^	
**Variables**			
Continuous, mean (SD)			
Age at index event	57.28 (12.65)	58.02 (11.62)	<.001
Charlson comorbidity index score	1.15 (1.81)	1.12 (1.76)	.04
Categorical			
SCS			
Yes	1725 (5.18)	3882 (4.95)	.10
No	31 554 (94.81)	74 585 (95.05)	
Sex			
Female	18 074 (54.31)	44 217 (56.35)	<.001
Male	15 194 (45.66)	34 235 (43.63)	
Race			
White	12 745 (38.30)	29 168 (37.17)	<.001
African American	1360 (4.09)	2671 (3.40)	
Asian	36 (0.11)	54 (0.07)	
Unknown	19 138 (57.51)	46 574 (59.35)	
Psychosocial history^c^			
Smoking	11 191 (33.63)	23 496 (29.94)	<.001
Alcohol abuse	1069 (3.21)	1674 (2.13)	<.001
Nonalcohol substance use disorder	7151 (21.49)	14 784 (18.84)	<.001
Long-term opioid use	33 279 (100.00)	78 467 (100.00)	NA
Depression diagnosis	8509 (25.57)	18 140 (23.12)	<.001
Anxiety diagnosis	6894 (20.72)	14 389 (18.34)	<.001
Psychosis diagnosis	721 (2.17)	1357 (1.73)	<.001
Current medications^c^			
Antidepressant use	15 757 (47.35)	42 570 (54.25)	<.001
Benzodiazepine use	14 657 (44.04)	37 020 (47.18)	<.001
Antipsychotic use	3119 (9.37)	7873 (10.03)	.001
Systemic comorbidities^d^			
Myocardial infarction	1393 (4.19)	3097 (3.95)	.07
Congestive heart failure	2125 (6.39)	4864 (6.20)	.24
Peripheral vascular disease	3007 (9.04)	6755 (8.61)	.02
Cerebrovascular disease	2932 (8.81)	6447 (8.22)	.001
Dementia	309 (0.93)	502 (0.64)	<.001
Chronic pulmonary disease	9153 (27.50)	21 262 (27.10)	.16
Rheumatic disease	1874 (5.63)	4510 (5.75)	.45
Peptic ulcer disease	996 (2.99)	2306 (2.94)	.64
Liver disease	2460 (7.39)	5228 (6.66)	<.001
Diabetes	7528 (22.62)	17 329 (22.08)	.05
Hemiplegia/paraplegia	663 (1.99)	1344 (1.71)	.001
Kidney disease	2154 (6.47)	4791 (6.11)	.02
Any malignant neoplasm	2029 (6.10)	4312 (5.50)	<.001
Metastatic solid tumor	543 (1.63)	1084 (1.38)	.002
AIDS/HIV	110 (0.33)	220 (0.28)	.18

Abbreviation: SCS, spinal cord stimulation.

^a^ Opioid-naive is defined as at most 2 prescriptions per year.

^b^ Long-term opioid use is defined as at least 6 prescriptions per year.

^c^ 1-year prior to index date.

^d^ Any time prior to index date.

**Figure 3.**
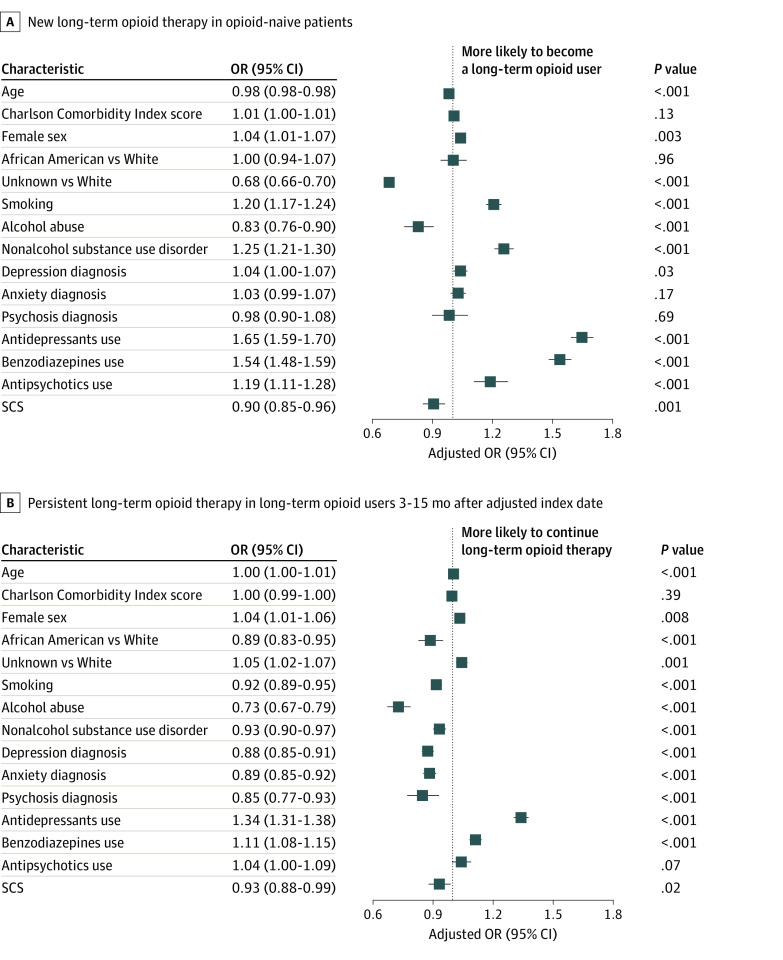
Multivariable Analysis Showing Factors Associated With New LOT in Opioid-Naive Patients and Persistent LOT in Long-term Opioid Users 3 to 15 Months After Adjusted Index Date (for Patients Without SCS) or SCS Implant in Diamond Network LOT was defined as at least 6 prescriptions per year. Opioid-naive was defined as at most 2 prescriptions per year. LOT indicates long-term opioid therapy; OR, odds ratio; SCS, spinal cord stimulation.

Figure 3 illustrates adjusted odd ratios based on multivariable logistic regression. Unlike in univariable analysis, Charlson comorbidity index score, anxiety, and psychosis diagnoses were not significantly associated with LOT for opioid-naive patients with PLS patients, whereas Charlson comorbidity index score and antipsychotic use were not significantly associated with LOT for patients with PLS previously on LOT. SCS was associated with a slightly increased likelihood of not being on opioids in both the opioid-naive (adjusted OR, 0.90 [95% CI, 0.85-0.96]; *P* < .001) and the LOT (adjusted OR, 0.93 [95% CI, 0.88-0.99]; *P* = .02) groups. This finding was replicated when the more stringent definition of LOT (≥10 opioid prescriptions per year) was applied, but not among opioid-naive patients using less stringent criterion (eFigure 4 in the Supplement).

### Associations Between Baseline Opioid Use, SCS, and Subsequent Opioid Use

For subsequent opioid use status, 370 841 patients were opioid-naive and 106 075 were on LOT. Patients with an SCS received a higher mean (SD) number of opioid prescriptions 1-year prior to implantation compared with those who did not receive an SCS (4.3 [8.5] prescriptions vs 4.1 [9.3] prescriptions; *P* < .001). However, the subsequent mean (SD) opioid prescriptions (3-15 months after the index date) were significantly lower in patients who received SCS vs those who did not (3.8 [8.2] prescriptions vs 4.0 [9.4] prescriptions; *P* = .006).

### Propensity Score Matching Analysis

In the propensity score matching analysis, SCS was associated with a statistically significant but clinically questionable reduction in LOT (OR, 0.93 [95% CI, 0.87-0.98]; *P* = .01) in the primary and more stringent analyses, but not by the most liberal definition of LOT (≥4 prescriptions per year) (OR, 0.94 [95% CI, 0.89-1.001]; *P* = .06). Among opioid-naive patients, SCS was also associated with a statistically significant but clinically questionable decreased odds of starting opioids when LOT was defined as at least 6 prescriptions per year (OR, 0.92 [95% CI, 0.87-0.98]; *P* = .01) and for the more stringent definition, but not when LOT was defined more loosely (OR, 0.98 [95% CI, 0.93-1.03]; *P* = .37) (eTable 5 in the Supplement).

## Discussion

This cohort study found that although SCS was associated with a small reduction in LOT at 3 to 15-month follow-up in both opioid-naive and primary LOT groups, the reduction was clinically questionable in the LOT group based on the propensity score matching analyses. Notably, a higher percentage of patients who underwent SCS implantation were previously on LOT than those who did not receive an SCS (26.0% vs 24.2%), suggesting that some pain physicians or patients may have trialed SCS as a means to reduce opioid intake.

Most studies touting beneficial effects of SCS enrolled a small number of highly selected patients, who were closely observed by research investigators. Accessing the health information of millions of patients with PLS using the TriNetX Diamond Network allowed us to assess the association between SCS and opioid use in the hundreds of thousands of patients with PLS, enhancing generalizability. Only 4.7% of patients with PLS received an SCS implant; this may reflect underutilization of this procedure, which has been shown in small, controlled studies to be effective for this condition and reduce opioid consumption.^11^ Alternatively, it may also reflect outcome differences between highly selective, industry-sponsored studies and nonindustry-sponsored trials, the latter of which may be more generalizable and less likely to demonstrate significant benefit.^6^ Our data also reveal that a disproportionate percentage of patients with PLS who received an SCS implant were White. This finding calls into question the accessibility of specialized health care to racial and ethnic minority populations in the US.

Reduction in analgesic consumption is an important outcome measure in an increasing number of studies evaluating the effectiveness of pain management interventions as it provides an objective measure of benefit. If an intervention is useful but not curative, many researchers believe it should reduce the need for opioids. We found that patients in the SCS group used significantly more opioids pre-SCS implantation or PLS diagnosis compared with those without an SCS, but significantly less post-SCS implantation or PLS index date, in multivariable analysis and with propensity score matching. However, in univariable analysis, no statistical or clinically relevant reduction in opioid use among patients on LOT was observed. Statistical interventions to control for possible population differences are based on assumptions that may or may not be true and are not testable in this context, and the differences observed even when controlling for possible confounding factors were small and may not have been clinically relevant. For example, some pain physicians may be reluctant to prescribe opioids to patients they have not treated and are not following, which could include those who decline neuromodulation. Among opioid-naive patients, SCS implantation was associated with a small reduction in subsequent opioid use when 6 and 10 prescriptions per year were used as cutoffs, but not when at least 4 prescriptions per year was used. This could theoretically reflect that more patients receiving SCS are intent on avoiding opioids at all costs, or are deemed to be poor candidates for LOT (eg, strong family history of substance abuse).

### Comparison With Other Studies

Several studies and reviews have examined the association between SCS and opioid use in patients with PLS, with most finding an opioid-sparing effect. In a small database review (n = 5476), Sharan et al^19^ found that those who continued SCS 1 year after implantation were able to slightly decrease their opioid consumption (from 98 morphine equivalents per day 1 month after SCS to 73 morphine equivalents per day at 12 months) compared with no significant opioid decrease in the 390 patients who were explanted within a year. In this study, most patients had either back pain, limb pain, or PLS. In a more recent database review involving 5878 patients, Fraifeld et al^16^ reported that 42% were able to discontinue or meaningfully decrease (≥50%) their opioid intake, although individuals with PLS (number not reported) were significantly less likely to do so. Our results are less propitious than those of Pollard et al^11^ who found a large opioid-sparing effect among patients on preimplant opioids in a meta-analysis involving 5 studies (n = 498), 4 of which were industry sponsored. However, they are more favorable than a database review involving 2374 patients who underwent SCS paddle lead placement for a variety of different indications including PLS, radiculopathy, and lumbago, that actually reported an increase in post-implant opioid consumption in 5 of the 8 years analyzed (with no statistical difference in the other 3 years).^20^ In this study, no pain comorbidity was significantly associated with a greater likelihood of reducing opioid consumption, and the criteria for defining pre- and postsurgical opioid consumption were more liberal than ours. It is important to note that there are no clinical trials evaluating pure mu opioid agonists for PLS in a nonaddicted population, and that opioids may exacerbate central sensitization in those who fail spine surgery with nociplastic, nonspecific spinal pain.^21,22^

### Explanation of Findings

There are several explanations for the discordance with studies reporting decreases in opioid consumption after SCS implantation.^11,23^ The present study reflects the association between opioid prescribing and SCS outside of a highly controlled research setting, wherein the selection criteria might be broader or even inappropriate and/or driven by financial considerations. A surprisingly high proportion of patients who received an SCS in our study had active psychopathology, which would constitute a contraindication in most industry-sponsored trials.^6,24^ Paradoxically, besides nonalcohol substance use disorder and depression, which were associated with LOT in previously opioid-naive patients, all other psychiatric comorbidities in our study were either associated with reduced opioid consumption or bore no association with post-surgical or post-implant LOT. It is also possible that patients with non-neuropathic spinal pain and/or those taking very high doses of opioids were more likely to receive SCS implants; such patients would be more likely to fail SCS and continue on LOT.^6,25,26^

### Limitations

Our study has several limitations. First, in addition to the flaws inherent in a retrospective cohort study, we could neither validate the diagnosis of PLS nor the appropriateness of SCS implantation. Second, although we included the most commonly used opioids in our analysis, some were excluded, such as those used to treat opioid misuse disorder (eg, methadone and buprenorphine, which are less frequently used solely for pain) and mixed action opioids (eg, tramadol and tapentadol, due to their lower risk profile, and for tramadol, because refills allow for multiple prescriptions to be given at a single visit).^27^ Third, our primary outcome measure was based on the number of opioid prescriptions, not dose, and we systematically excluded patients receiving intermittent opioid therapy. Therefore, it is conceivable that some patients who received an SCS were able to reduce but not eliminate their opioid consumption, and that SCS may be associated with an increased likelihood of opioid discontinuation in individuals receiving periodic prescriptions. Fourth, although opioid prescriptions and consumption are highly correlated in a non-diverting population, we could only measure the former, which served as a surrogate for the latter. Finally, it was impossible to perform a subgroup analysis based on the different stimulation programs and lead configurations. However, previous studies have yielded conflicting results as to whether newer modalities are more effective than conventional ones, and studies comparing the two may be confounded by bias.^6,28^

## Conclusions

In summary, among all patients, SCS for PLS resulted in statistically significant reductions in the number of opioid prescriptions in some comparisons, but the reduction was small and its clinical relevance is questionable. However, SCS was associated with a lower rate of new opioid use in patients who were previously opioid-naive. Since one of the motivations to offer SCS to patients with PLS is to decrease or discontinue opioid use, further study is needed to evaluate this objective outcome measurement.

## Notes

Supplement. **eTable 1.** A List of ICD and CPT Codes Used to Extract Post-Laminectomy Syndrome Diagnosis, Spine Surgery Procedure, Spinal Cord Stimulation Device, and Other Relevant Clinical Diagnoses **eTable 2.** List of Opioid and Psychotropic Medications Used With RxNorm Codes Included in Parentheses **eTable 3.** List of ICD-9/10 Codes Used to Calculate Charlson Comorbidity Index Score **eTable 4.** Characteristics of Post-Laminectomy Syndrome Patients in Diamond Network Stratified by SCS Status **eTable 5.** Propensity Score Matching Analysis With Average Treatment Effect (ATE) Defined by Odds Ratios **eFigure 1.** Flowchart Illustrating the Inclusion and Exclusion Criteria of Postlaminectomy Syndrome Patients With and Without Spinal SCS **eFigure 2.** Multivariable Analysis Showing Factors Associated With Receiving an SCS Implant in PLS Patients **eFigure 3.** Breakdown of PLS Cohort by SCS Status, Baseline Opioid Status, and Opioid Use Status at 3-15 Months After Adjusted Index Date (for Patients Without SCS) or SCS Implant in Diamond Network **eFigure 4.** Multivariable Analysis With Sensitivity Analyses Showing Factors Associated With New COT in Opioid-Naive Patients (Left Panel) and Persistent COT in Chronic Opioid Users (Right Panel) 3-15 Months After Adjusted Index Date (for Patients Without SCS) or SCS Implant in Diamond Network
