# Supplementary material for: Association of Spinal Cord Stimulator Implantation With Persistent Opioid Use in Patients With Postlaminectomy Syndrome
Source: JAMA Netw Open. 2022 Jan 31;5(1):e2145876. doi: 10.1001/jamanetworkopen.2021.45876 (PMC8804916; doi:10.1001/jamanetworkopen.2021.45876)
Supplement: Supplement. — eTable 1. A List of ICD and CPT Codes Used to Extract Post-Laminectomy Syndrome Diagnosis, Spine Surgery Procedure, Spinal Cord Stimulation Device, and Other Relevant Clinical Diagnoses eTable 2. List of Opioid and Psychotropic Medications Used With RxNorm Codes Included in Parentheses eTable 3. List of ICD-9/10 Codes Used to Calculate Charlson Comorbidity Index Score eTable 4. Characteristics of Post-Laminectomy Syndrome Patients in Diamond Network Stratified by SCS Status eTable 5. Propensity Score Matching Analysis With Average Treatment Effect (ATE) Defined by Odds Ratios eFigure 1. Flowchart Illustrating the Inclusion and Exclusion Criteria of Postlaminectomy Syndrome Patients With and Without Spinal SCS eFigure 2. Multivariable Analysis Showing Factors Associated With Receiving an SCS Implant in PLS Patients eFigure 3. Breakdown of PLS Cohort by SCS Status, Baseline Opioid Status, and Opioid Use Status at 3-15 Months After Adjusted Index Date (for Patients Without SCS) or SCS Implant in Diamond Network eFigure 4. Multivariable Analysis With Sensitivity Analyses Showing Factors Associated With New COT in Opioid-Naive Patients (Left Panel) and Persistent COT in Chronic Opioid Users (Right Panel) 3-15 Months After Adjusted Index Date (for Patients Without SCS) or SCS Implant in Diamond Network [file jamanetwopen-e2145876-s001.pdf]

Supplemental Online Content

Vu TN, Khunsriraksakul C, Vorobeychik Y, et al. Association of spinal cord stimulator implantation with persistent opioid use in patients with postlaminectomy syndrome. *JAMA Netw Open.* 2022;5(1):e2145876. doi:10.1001/jamanetworkopen.2021.45876

eTable 1. A List of ICD and CPT Codes Used to Extract Post-Laminectomy Syndrome Diagnosis, Spine Surgery Procedure, Spinal Cord Stimulation Device, and Other Relevant Clinical Diagnoses

eTable 2. List of Opioid and Psychotropic Medications Used With RxNorm Codes Included in Parentheses

eTable 3. List of ICD-9/10 Codes Used to Calculate Charlson Comorbidity Index Score

eTable 4. Characteristics of Post-Laminectomy Syndrome Patients in Diamond Network Stratified by SCS Status

eTable 5. Propensity Score Matching Analysis With Average Treatment Effect (ATE) Defined by Odds Ratios

eFigure 1. Flowchart Illustrating the Inclusion and Exclusion Criteria of Postlaminectomy Syndrome Patients With and Without Spinal SCS

eFigure 2. Multivariable Analysis Showing Factors Associated With Receiving an SCS Implant in PLS Patients

eFigure 3. Breakdown of PLS Cohort by SCS Status, Baseline Opioid Status, and Opioid Use Status at 3-15 Months After Adjusted Index Date (for Patients Without SCS) or SCS Implant in Diamond Network

eFigure 4. Multivariable Analysis With Sensitivity Analyses Showing Factors Associated With New COT in Opioid-Naïve Patients (Left Panel) and Persistent COT in Chronic Opioid Users (Right Panel) 3-15 Months After Adjusted Index Date (for Patients Without SCS) or SCS Implant in Diamond Network

This supplemental material has been provided by the authors to give readers additional information about their work.

© 2022 Vu TN et al. *JAMA Network Open.*

eTable 1: A list of ICD and CPT codes used to extract post-laminectomy syndrome diagnosis, spine surgery procedure, spinal cord stimulation device, and other relevant clinical diagnoses.

Diagnosis	ICD-9 codes	ICD-10 codes	CPT codes	RxNorms
**Main**				
Post-laminectomy syndrome	722.80-722.83	M96.1	-	-
Spine surgery	-	-	22010-22899	-
Spinal cord stimulation	-	-	63655, 63663, 63664, 63685	-
**Psychosocial history**				
Smoking	305.1, 649.0, 989.84, V15.82	F17, O99.33, T65.22, Z71.6, Z72.0, Z87.891	99406, 99407, G0436, G0437, G9016, S9453, S4995, G9276, G9458	Nicotine (7407), Varenicline (591622)
Alcohol abuse	94.46, 94.53, 94.6, 265.2, 291, 303, 305.0, 357.5, 425.5, 535.3, 571.0-571.3, 760.71, 980.0, V11.3	E52, F10, G62.1, G72.1, I42.6, K29.2, K70, K85.2, K86.0, O99.31, T51.0X, Z71.4	-	-
Non-alcohol substance use disorder	292, 304, 305.2-305.9, V65.42	F11-F19	-	-
Depression diagnosis	296.2, 296.3, 296.5, 300.4, 309, 311	F31-F33, F34.1, F43	-	-
Anxiety diagnosis	300.0, 300.2	F40-F41	-	-
Psychosis diagnosis	293.8, 295, 296.04, 296.14, 296.44, 296.54, 297, 298	F06, F20, F22-F25, F28-F29, F30.2, F31.2, F31.5, F32.3, F33.3, F44.89	-	-

Abbreviations: ICD-9/10 = International Classification of Diseases, Ninth/Tenth Revision. CPT = Current Procedural Terminology.

© 2022 Vu TN et al. *JAMA Network Open.*

eTable 2: List of opioid and psychotropic medications used with RxNorm codes included in parentheses.

**Opioids**	
	Hydromorphone (3423), Hydrocodone (5489), Fentanyl (4337), Morphine (7052), Oxycodone (7804), Oxymorphone (7814)
	**Exclude:** Hydromorphone (3423) records with Guaifenesin (5032)<br>**Exclude:** Hydrocodone (5489) records with Guaifenesin (5032), Pseudoephedrine (8896), Homatropine (27084), Chlorpheniramine (2400)
**Benzodiazepines**	
	Alprazolam (596), Chlordiazepoxide (2356), Clonazepam (2598), Clorazepate (2353), Diazepam (3322), Estazolam (4077), Flurazepam (4501), Halazepam (26412), Lorazepam (6470), Midazolam (6960), Oxazepam (7781), Quazepam (35185), Temazepam (10355), Triazolam (10767)
**Antidepressants**	
SSRI	Citalopram (2556), Escitalopram (321988), Paroxetine (32937), Fluoxetine (4493), Fluvoxamine (42355), Sertraline (36437)
SNRI	Desvenlafaxine (734064), Duloxetine (72625), Levomilnacipran (1433212), Milnacipran (588250), Venlafaxine (39786)
SMS	Vilazodone (1086769), Vortioxetine (1455099)
SARI	Trazodone (10737), Nefazodone (31565)
TCA	Amitriptyline (704), Clomipramine (2597), Desipramine (3247), Imipramine (5691), Nortriptyline (7531), Protriptyline (8886), Trimipramine (10834)
TeCA	Amoxapine (722), Maprotiline (6646), Mirtazapine (15996)
MAOI	Isocarboxazid (6011), Phenelzine (8123), Tranylcypromine (10734), Selegiline (9639)
**Antipsychotics**	
Typical	Chlorpromazine (2403), Thioridazine (10502), Mesoridazine (6779), Loxapine (6475), Molindone (7019), Perphenazine (8076), Thiothixene (10510), Trifluoperazine (10800), Haloperidol (5093), Fluphenazine (4496), Droperidol (3648), Prochlorperazine (8704)
Atypical	Aripiprazole (89013), Asenapine (784649), Clozapine (2626), Iloperidone (73178), Lurasidone (1040028), Olanzapine (61381), Paliperidone (679314), Quetiapine (51272), Risperidone (35636), Ziprasidone (115698)

Abbreviations: SSRI = selective serotonin reuptake inhibitor, SNRI = serotonin-norepinephrine reuptake inhibitor, SMS = serotonin modulator and stimulator, SARI = serotonin antagonist and reuptake inhibitor, TCA = tricyclic antidepressant, TeCA = tetracyclic antidepressant, MAOI = monoamine oxidase inhibitor

© 2022 Vu TN et al. *JAMA Network Open.*

eTable 3: List of ICD-9/10 codes used to calculate Charlson Comorbidity Index score.

Comorbidity	ICD-9 codes	ICD-10 codes
Myocardial infarction	410, 412	I21-I22, I25.2
Congestive heart failure	398.91, 402.01, 402.11, 402.91, 404.01, 404.03, 404.11, 404.13, 404.91, 404.93, 425.4-425.9, 428	I09.9, I11.0, I13.0, I13.2, I25.5, I42.0, I42.5-I42.9, I43, I50, P29.0
Peripheral vascular disease	093.0, 437.3, 440-441, 443.1-443.9, 447.1, 557.1, 557.9, V43.4	I70-I71, I73.1, I73.8, I73.9, I77.1, I79.0, I79.2, K55.1, K55.8, K55.9, Z95.8, Z95.9
Cerebrovascular disease	362.34, 430-438	G45-G46, H34.0, I60-I69
Dementia	290, 294.1, 331.2	F00-F03, F05.1, G30, G31.1
Chronic pulmonary disease	416.8, 416.9, 490-505, 506.4, 508.1, 508.8	I27.8-I27.9, J40-J47, J60-J67, J68.4, J70.1, J70.3
Rheumatic disease	446.5, 710.0-710.4, 714.0-714.2, 714.8, 725	M05-M06, M31.5, M32-M34, M35.1, M35.3, M36.0
Peptic ulcer disease	531-534	K25-K28
Mild liver disease	070.22-070.23, 070.32-070.33, 070.44, 070.54, 070.6, 070.9, 570-571, 573.3-573.4, 573.8-573.9, V42.7	B18, K70.0-K70.3, K70.9, K71.3-K71.5, K71.7, K73-K74, K76.0, K76.2-K76.4, K76.8-K76.9, Z94.4
Diabetes without chronic complication	250.0-250.3, 250.8, 250.9	E10.0-E10.1, E10.6, E10.8-E11.1, E11.6, E11.8-E12.1, E12.6, E12.8-E13.1, E13.6, E13.8-E14.1, E14.6, E14.8-E14.9
Diabetes with chronic complication	250.4-250.7	E10.2-E10.5, E10.7, E11.2-E11.5, E11.7, E12.2-E12.5, E12.7, E13.2-E13.5, E13.7, E14.2-E14.5, E14.7
Hemiplegia or paraplegia	334.1, 342-344.6, 344.9	G04.1, G11.4, G80.1, G80.2, G81-G83.4, G83.9
Renal disease	403.01, 403.11, 403.91, 404.02-404.03, 404.12-404.13, 404.92-404.93, 582-583.7, 585-586, 588.0, V42.0, V45.1, V56	I12.0, I13.1, N03.2-N03.7, N05.2-N05.7, N18-N19, N25.0, Z49.0-Z49.2, Z94.0, Z99.2
Any malignancy, including lymphoma and leukemia (except malignant neoplasm of skin)	140-172, 174-195.8, 200-208, 238.6	C00-C26, C30-C34, C37-C41, C43, C45-C58, C60-C76, C81-C85, C88, C90-C97
Moderate or severe liver disease	456.0-456.2, 572.2-572.8	I85.0, I85.9, I86.4, I98.2, K70.4, K71.1, K72.1, K72.9, K76.5-K76.7
Metastatic solid tumor	196-199	C77-C80
AIDS/HIV	042-044	B20-B22, B24

Abbreviations: ICD-9/10 = International Classification of Diseases, Ninth/Tenth Revision

© 2022 Vu TN et al. *JAMA Network Open.*

eTable 4: Characteristics of post-laminectomy syndrome patients in Diamond Network stratified by SCS status.

Characteristic	No SCS (N = 526,758)	SCS (N = 26,179)	P-value
**Continuous variables**	**Mean ± SD**	**Mean ± SD**	
Age at index event (mean, SD)	59.77+12.72	61.38+12.94	**< 0.001**
Charlson score (mean, SD)	1.15+1.79	1.21+1.80	**< 0.001**
**Categorical variables**	**Number (%)**	**Number (%)**	
Sex (number, %)			**< 0.001**
Female	291,002 (55.24)	14,800 (56.53)	
Male	235,592 (44.72)	11,369 (43.43)	
Race (number, %)			**< 0.001**
White	176,600 (33.53)	8,539 (32.62)	
African American	18,412 (3.50)	559 (2.14)	
Asian	633 (0.12)	15 (0.06)	
Unknown	331,113 (62.86)	17,066 (65.19)	
Psychosocial history (number, %)*			
Smoking	122,403 (23.24)	6,908 (26.39)	**< 0.001**
Alcohol abuse	11,054 (2.10)	496 (1.89)	**0.023**
Non-alcohol substance use disorder	70,447 (13.37)	3,916 (14.96)	**< 0.001**
Chronic opioid use	117,328 (22.27)	6,225 (23.78)	**< 0.001**
Depression diagnosis	102,476 (19.45)	7,393 (28.24)	**< 0.001**
Anxiety diagnosis	79,318 (15.06)	4,876 (18.63)	**< 0.001**
Psychosis diagnosis	8,912 (1.69)	530 (2.02)	**< 0.001**
Current medications (number, %)*			
Antidepressant use	125,051 (23.74)	7,419 (28.34)	**< 0.001**
Benzodiazepine use	107,029 (20.32)	6,274 (23.97)	**< 0.001**
Antipsychotic use	21,666 (4.11)	1,207 (4.61)	**< 0.001**
Systemic comorbidities (number, %)**			
Myocardial infarction	19,621 (3.72)	1,274 (4.87)	**<0.001**
Congestive heart failure	31,741 (6.03)	1,921 (7.34)	**<0.001**
Peripheral vascular disease	48,863 (9.28)	3,235 (12.36)	**<0.001**
Cerebrovascular disease	46,179 (8.77)	2,833 (10.82)	**<0.001**
Dementia	4,377 (0.83)	295 (1.13)	**<0.001**
Chronic pulmonary disease	129,155 (24.52)	7,599 (29.03)	**<0.001**
Rheumatic disease	28,387 (5.39)	1,767 (6.75)	**<0.001**
Peptic ulcer disease	13,404 (2.54)	885 (3.38)	**<0.001**
Liver disease	33,613 (6.38)	2,013 (7.69)	**<0.001**
Diabetes	121,138 (23.00)	7,174 (27.40)	**<0.001**
Hemiplegia/paraplegia	9,400 (1.78)	484 (1.85)	0.46
Renal disease	35,190 (6.68)	2,424 (9.26)	**<0.001**
Any malignancy	33,010 (6.27)	1,888 (7.21)	**<0.001**
Metastatic solid tumor	6,359 (1.21)	325 (1.24)	0.64
AIDS/HIV	1,596 (0.30)	83 (0.32)	0.73

*1-year prior to index date
**Any time prior to index date

© 2022 Vu TN et al. *JAMA Network Open.*

eTable 5: Propensity score matching analysis with average treatment effect (ATE) defined by odds ratios.

Chronic opioid use definition	Prior opioid use	Before matching		After matching		OR [95% CI]	P-value
		#Controls	#Cases	#Controls	#Cases		
≥4	No	358,771	17,335	173,328	17,334	0.977 [0.927, 1.029]	0.37
≥4	Yes	113,422	6,013	60,094	6,013	0.944 [0.890, 1.001]	0.06
≥6	No	348,403	16,767	167,636	16,766	0.923 [0.867, 0.982]	**0.01**
≥6	Yes	106,139	5,607	56,034	5,607	0.926 [0.873, 0.983]	**0.01**
≥10	No	337,491	16,256	162,539	16,255	0.874 [0.805, 0.948]	**0.001**
≥10	Yes	91,465	4,783	47,780	4,783	0.925 [0.869, 0.984]	**0.01**

© 2022 Vu TN et al. *JAMA Network Open.*

eFigure 1: Diamond Network demographics. Flow chart illustrating the inclusion and exclusion criteria of post-laminectomy syndrome patients with and without spinal SCS from TriNetX Diamond Network. For cases, we defined the index date as the date of SCS implantation. For controls, we randomly assigned an index date after PLS diagnosis that matches the distribution of days between PLS diagnosis and SCS placement in the case group.

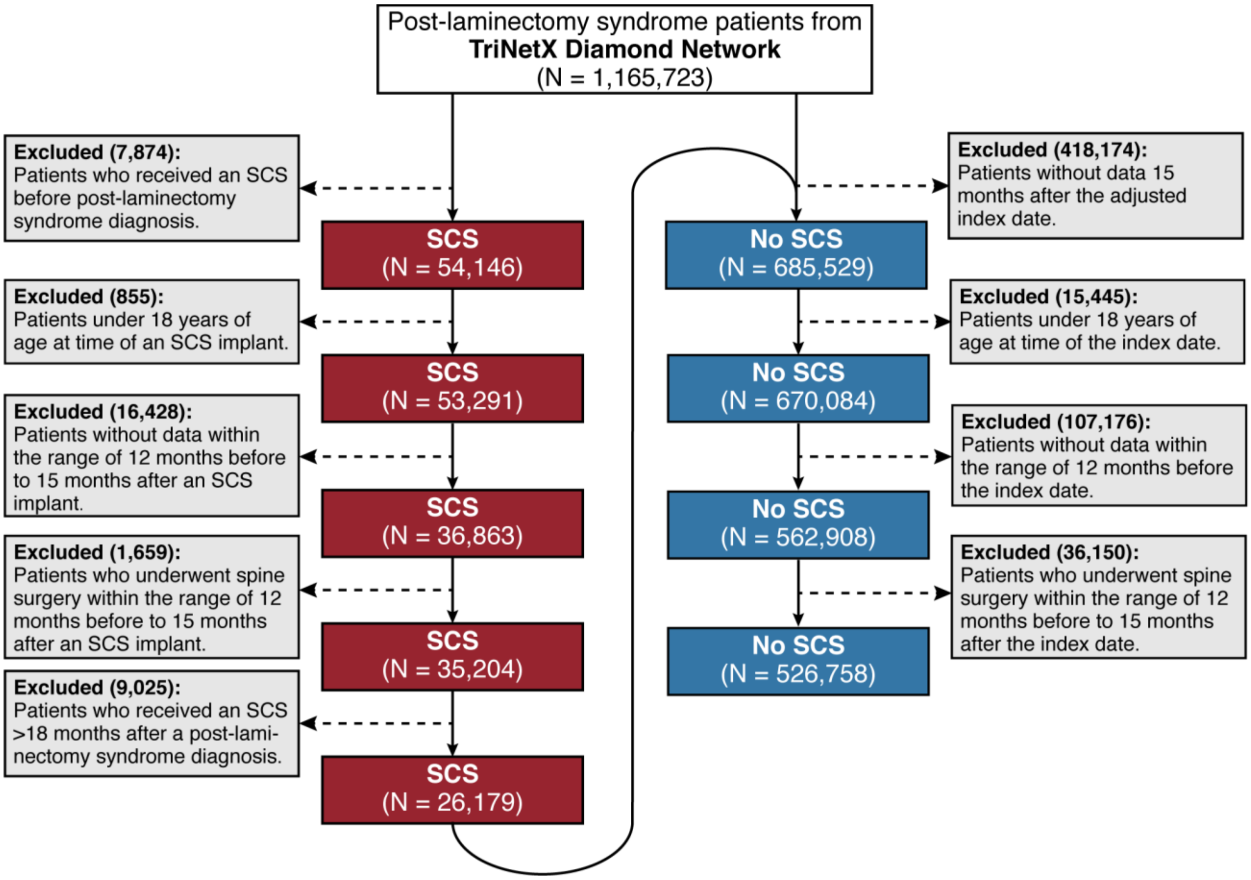

PLS- post-laminectomy syndrome; SCS- spinal cord stimulation

© 2022 Vu TN et al. *JAMA Network Open.*

eFigure 2: Multivariable analysis showing factors associated with receiving an SCS implant in PLS patients.

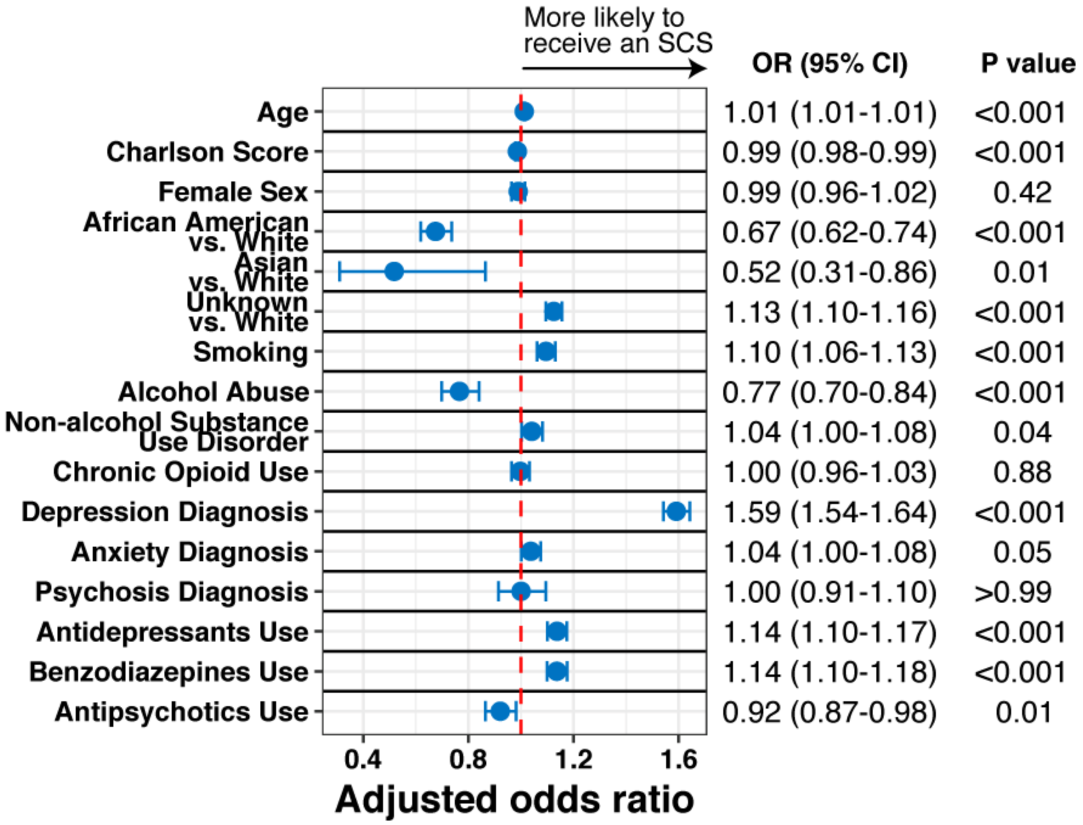

PLS- post-laminectomy syndrome; SCS- spinal cord stimulation

© 2022 Vu TN et al. *JAMA Network Open.*

eFigure 3: Breakdown of PLS cohort by SCS status, baseline opioid status, and opioid use status at 3-15 months after adjusted index date (for patients without SCS) or SCS implant in Diamond Network. (**A**) Chronic opioid therapy is defined as ≥4 prescriptions/year. (**B**) Chronic opioid therapy is defined as ≥10 prescriptions/year.

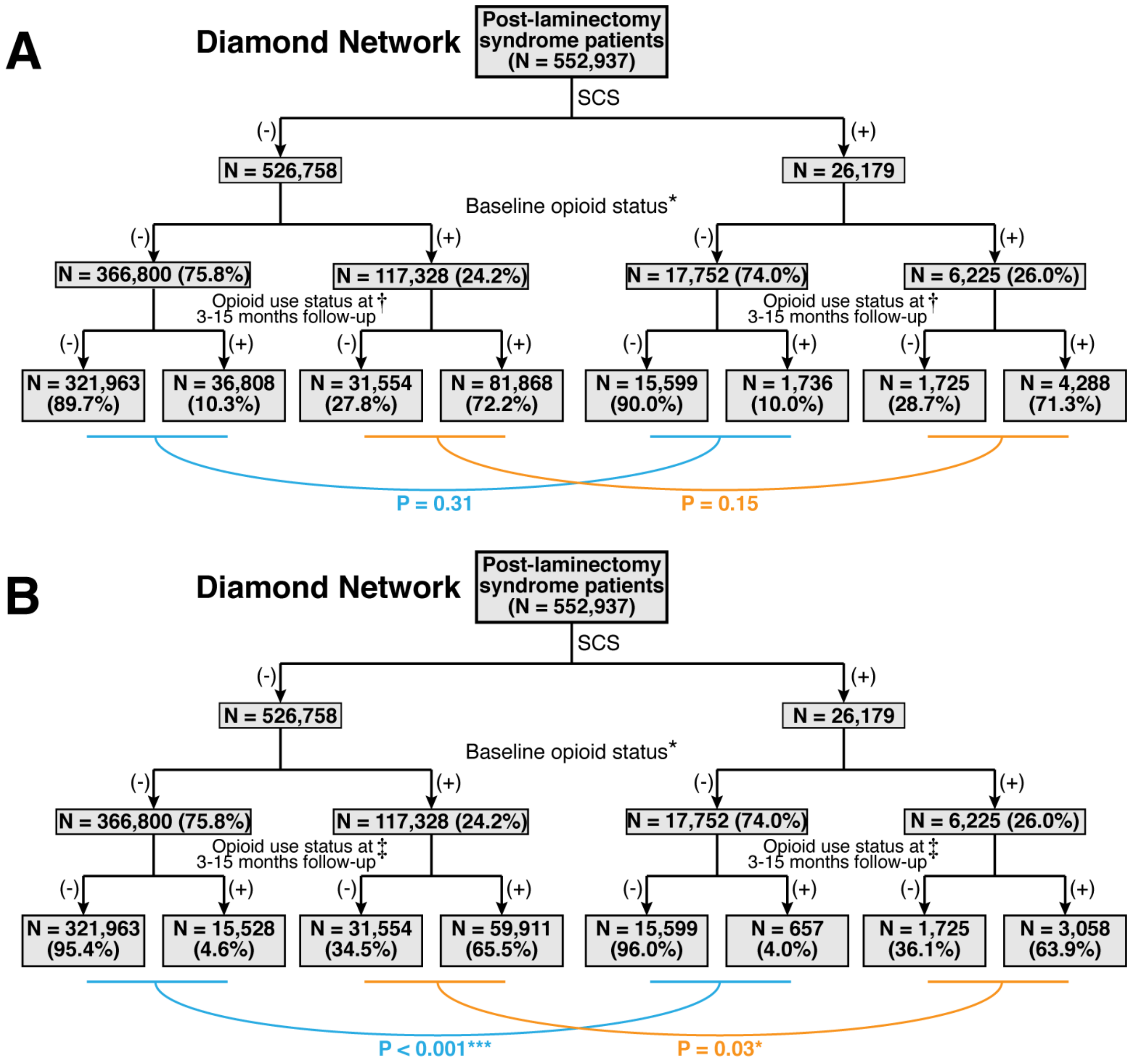

* Baseline opioid status: (+) is defined as ≥6 prescriptions/year and (-) is defined as ≤2 prescriptions/year.
† Opioid use status: (+) is defined as ≥4 prescriptions/year and (-) is defined as ≤2 prescriptions/year.
‡ Opioid use status: (+) is defined as ≥10 prescriptions/year and (-) is defined as ≤2 prescriptions/year.

PLS- post-laminectomy syndrome; SCS- spinal cord stimulation

© 2022 Vu TN et al. *JAMA Network Open.*

eFigure 4: Multivariable analysis with sensitivity analyses showing factors associated with new COT in opioid-naïve patients (left panel) and persistent COT in chronic opioid users (right panel) 3-15 months after adjusted index date (for patients without SCS) or SCS implant in Diamond Network. Chronic opioid use is defined as ≥4, ≥6, or ≥10 prescriptions/year, while opioid-naïve is defined as ≤2 prescriptions/year.

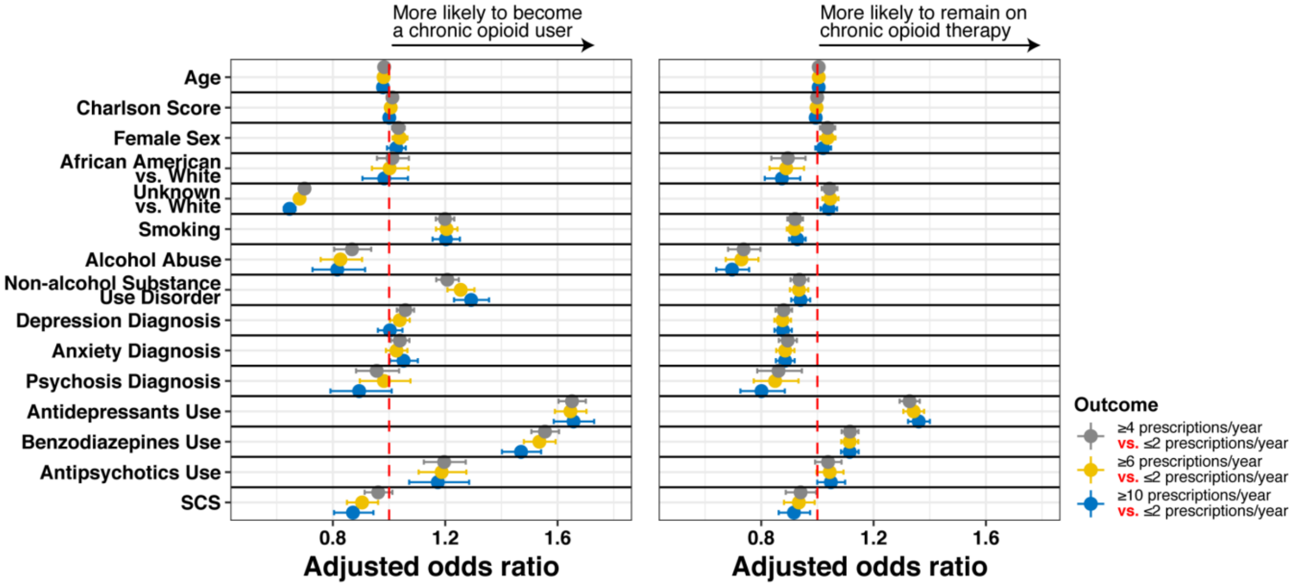

COT- chronic opioid therapy; SCS- spinal cord stimulation

© 2022 Vu TN et al. *JAMA Network Open.*
